# Successful Sperm Retrieval and Clinical Pregnancies Following Micro-TESE and ICSI Treatments in Patients with Nonobstructive Azoospermia Due to Various Etiologies

**DOI:** 10.3390/cells13181582

**Published:** 2024-09-20

**Authors:** Guangmin Liu, Zenghui Huang, Wenbing Zhu, Huan Zhang, Liqing Fan, Chuan Huang

**Affiliations:** 1 NHC Key Laboratory of Human Stem Cell and Reproductive Engineering, School of Basic Medical Sciences, Central South University, Changsha 410017, China; liuguangmin@csu.edu.cn (G.L.); zhuwenbing0971@sina.com (W.Z.); 2 Clinical Research Center for Reproduction and Genetics in Hunan Province, Reproductive & Genetic Hospital of CITIC-Xiangya, Changsha 410008, China; hzhjt2018@163.com

**Keywords:** non-obstructive azoospermia, microdissection testicular sperm extraction, sperm retrieval rate, pregnancy rate

## Abstract

(1) Background: Nonobstructive azoospermia (NOA) etiologies affect the sperm retrieval rate (SRR) by microdissection testicular sperm extraction (micro-TESE) and the clinical outcomes following intracytoplasmic sperm injection (ICSI); (2) Methods: We investigated seven NOA etiologies. The SRR and clinical outcomes of 627 patients were analyzed between November 2017 and July 2022 in the Reproductive and Genetic Hospital of China International Trust and Investment Corporation-Xiangya (CITIC-Xiangya); (3) Results: The overall SRR was 39.4% (247/627). The SRR according to NOA etiologies were: Y chromosome azoospermia factor c microdeletions (26/46, 56.5%), Klinefelter syndrome (KS), 36/85, 42.4%), idiopathic (110/398, 27.6%), cryptorchidism (20/29, 69.0%), chromosome anomalies (7/13, 53.9%), orchitis (45/50, 90.0%), and cancer (3/6, 50.0%). The SRR were different for spermatogonia arrest (26/96, 27.1%), maturation arrest (76/177, 42.9%), and SCOS (30/80, 37.5%) according to histological examinations. The clinical pregnancy rate was similar among the NOA etiologies. The high-quality embryo rate differed between successful (54.7%) and unsuccessful (40.9%) pregnancies. Moreover, the successfully pregnant women (28.99 years) were younger than the unsuccessfully pregnant ones (30.92 years); (4) Conclusions: The SRR from patients with NOA was associated with the etiology and histological categories, while the clinical outcome was associated with the high-quality embryo rate and the female partner’s age.

## 1. Introduction

Infertility is a major health problem worldwide and is estimated to affect 8–12% of couples in the reproductive age group, with the male factor being a primary or contributing cause in approximately 50% of couples [1]. NOA, defined as the absence of spermatozoa in the ejaculate following centrifugation, is the most severe form of male infertility, occurring in approximately 1% of men and 10% of infertile men [2]. While the etiology of most (60–70%) NOA cases is idiopathic, some well-known etiologies include KS, Y-chromosome microdeletions (YCMDs), malignancies, cryptorchidism, chromosome anomalies, and orchitis [3]. NOA remains a challenge for andrologists who aim to help patients father their biological children. With the development of human-assisted reproductive technologies, NOA patients could father their biological offspring if normal sperm were found in their testes. Therefore, successful sperm retrieval is especially significant for patients with NOA.

Testicular spermatozoa can be recovered in some patients with NOA because some isolated foci of active spermatogenesis are present in their testes. Several techniques have been described for spermatozoa retrieval in these patients, including ultrasound-guided fine needle aspiration, conventional testicular sperm extraction (c-TESE), and micro-TESE [4]. Micro-TESE, introduced by Schlegel in 1999, allows magnifying the testis parenchyma under an operating microscope to select the whitish, larger, and opaque tubules that are more likely to contain spermatozoa [5]. This standard technique is likely to result in a high SRR with minimal tissue loss. ICSI with micro-TESE is commonly used for patients with NOA. However, most previous studies focused on sperm extraction techniques and showed that cTESE/mTESE in subjects with NOA results in SRRs of up to 50% [6]. A few studies provided data on the SRR and clinical outcomes of patients with NOA due to various etiologies [7,8] and the retrieved sperm quantity after micro-TESE. The factors affecting clinical pregnancies in couples whose male partner has NOA remain unclear. This study aimed to analyze the SRR, clinical pregnancy rate (CPR), and the factors affecting these following micro-TESE and ICSI treatments in patients with NOA due to various etiologies and their partners.

## 2. Materials and Methods

### 2.1. Patients

**Figure 1:**
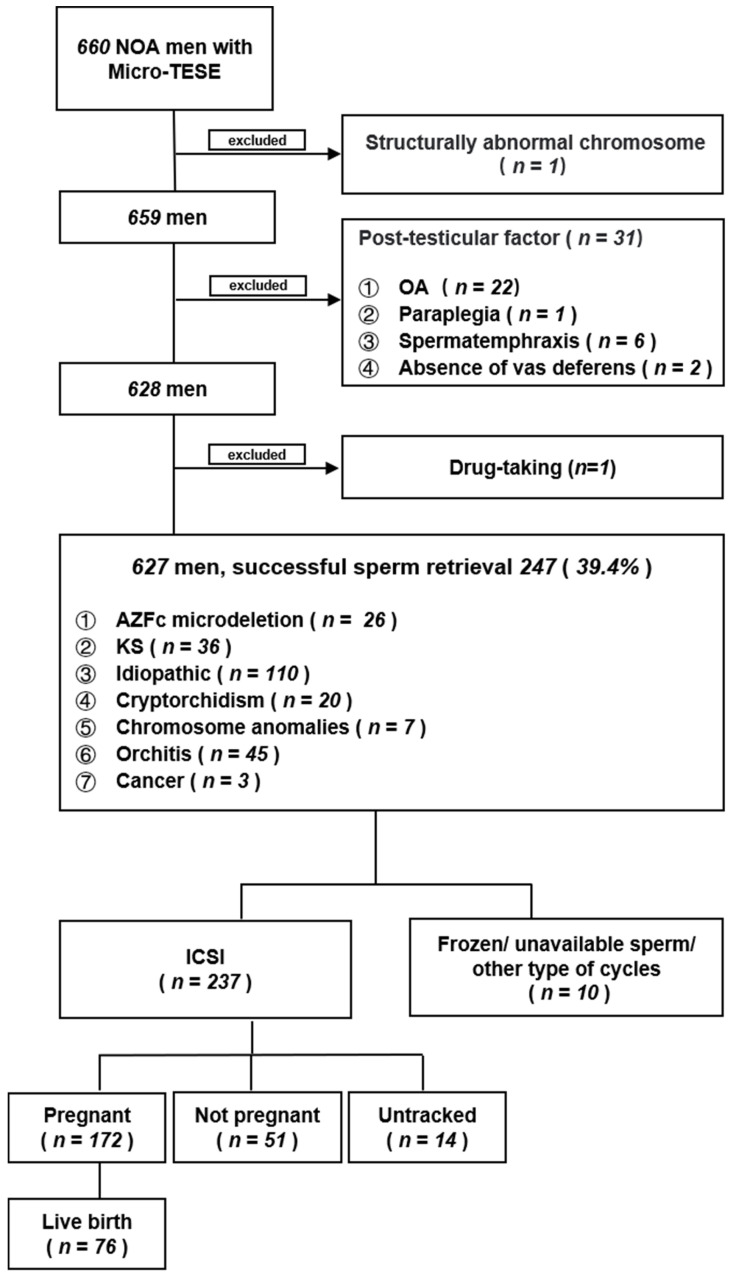
Schematic overview of this study. Abbreviations: NOA, non-obstructive azoospermia; Micro-TESE, microdissection testicular sperm extraction; OA, obstructive azoospermia; AZFc, Y chromosome azoospermia factor c; KS, Klinefelter syndrome; ICSI, intracytoplasmic sperm injection.

This retrospective study included 660 men who underwent micro-TESE in the Reproductive and Genetic Hospital of CITIC-Xiangya between November 2017 and July 2022. We excluded 33 patients with normal spermatogenesis in the testes but showed azoospermia due to structurally abnormal chromosomes, post-testicular factors (OA, paraplegia, spermatemphraxis, and absence of vas deferens), and the use of drugs. The data of 627 patients presenting with NOA were analyzed (Figure 1).

NOA was confirmed by at least three semen analyses with no spermatozoa after centrifugation at 3000× *g* for 15 min. The patients underwent a complete clinical evaluation to determine the etiology of azoospermia, including clinical history, physical examination, testicular ultrasonography, sex hormone evaluation [follicle-stimulating hormone (FSH), luteinizing hormone (LH), testosterone (T), estradiol (E2), and prolactin (PRL)], karyotyping, and YCMDs analysis. The female partners have healthy fertility in this study.

The Ethics Committee of the Reproductive and Genetic Hospital of CITIC-Xiangya approved the present study (LL-SC-2020-005). Participants in this study were informed and consented.

### 2.2. Micro-TESE

After extracting the testis through a scrotal wall incision, a transverse incision in the tunica was made to expose the testicular parenchyma. The micro-TESE was performed by two surgeons in the binocular operating microscope (OPMI LUMERA^®^ 700, Carl Zeiss Meditec AG, Oberkochen, Germany) simultaneously. And the seminiferous tubules were cut into pieces in sterile human tubal fluid (HTF) solution and investigated under an operating microscope by a technician from Human Sperm Bank. Only dilated tubules were biopsied and examined for spermatozoa. Dissection and biopsy were continued until an adequate number of spermatozoa were retrieved. The procedure was repeated on the other testicle if no spermatozoa could be retrieved from the first one. If no dilated tubules were encountered, multiple random biopsy samples from all testicular compartments were taken and examined by histopathology.

### 2.3. Histopathology Data

According to the document of pathology reports, the histopathological results were divided into three types, including spermatogonia arrest, maturation arrest, and SCOS. Spermatogonia arrest is defined as having only spermatogonium cells or fewer spermatogonium cells. Maturation arrest is defined as there is no complete spermatogenesis and no sperm in the spermatogenic tubules. SCOS is defined as there being only Sertoli cells in the spermatogenic tubules.

#### Ovarian Stimulation and Oocyte Retrieval

The ovarian stimulation protocol combined gonadotrophin-releasing hormone (GnRH) analogs (Gonal-f, Merck Serono, Darmstadt, Germany), FSH (Gonal-f, Merck Serono, Darmstadt, Germany), and human chorionic gonadotrophin (hCG, Gonal-f, Merck Serono, Darmstadt, Germany). Oocytes were retrieved by vaginal ultrasound-guided puncture 36–38 h after hCG administration. The obtained oocytes were maintained in insemination liquid in a 37 °C incubator with 5% CO_2_, 5% O_2_, and 90% N_2_. Cumulus cells were removed by pipetting and exposed to hyaluronidase 2 h after retrieval [7].

### 2.4. ICSI and Embryo Transfer

ICSI was performed as described in detail elsewhere [9]. Fertilization was assessed by the presence of two pronuclei and two polar bodies 17–19 h after insemination. On day 3, at the cleavage stage, a well-developed embryo is defined as more than 7 cells with a fragmentation rate of less than 10% and no vacuoles. At the blastula stage, according to the Gardner criterion, a well-developed embryo is defined as one whose blastocyst grade is greater than 3BB. Well-developed morulas or blastocysts were selected for transfer. The number of transferred embryos was usually limited to two to reduce the risk of multiple pregnancies.

### 2.5. Statistical Analysis

Statistical analysis was performed using GraphPad Prism 8 (GraphPad Software Inc., San Diego, CA, USA). Continuous normally distributed variables are expressed as mean ± SEM or *n* (%) and were analyzed by the student’s *t*-test. In cases of unequal variances or non-Gaussian distribution, the Welch’s correction or Mann-Whitney U test was used, respectively. One-way analysis of variance (ANOVA) compared data among more than two groups. Chi-squared or Fisher’s exact test assessed proportions. The odds ratio (OR) and 95% confidence interval (CI) of the multivariate associations between female age, body mass index (BMI), anti-Müllerian hormone (AMH), NOA etiologies, and clinical pregnancy were assessed using binary logistic regression models by SPSS 26 (SPSS Inc., Chicago, IL, USA). Statistical significance was set at *p* < 0.05.

## 3. Results

### 3.1. NOA Cohort Patients’ Characteristics

During this study period, 660 patients with NOA underwent micro-TESE. We excluded 33 patients with normal spermatogenesis in the testes and analyzed 627. Sperm was successfully retrieved in 247/627 (39.4%) patients (Figure 1). Baseline characteristics, including age, BMI, FSH, LH, T, PRL, inhibin B, and left and right testis volumes, did not differ between those with and without successful sperm retrieval (Table 1).

### 3.2. Micro-TESE SRR in Patients with NOA Due to Various Etiologies

The overall micro-TESE SRR was 39.4% (247/627). The SRR showed differences among etiologies, including Y chromosome azoospermia factor c (AZFc) microdeletions (26/46, 56.5%), KS (36/85, 42.4%), idiopathic (110/398, 27.6%), cryptorchidism (20/29, 69.0%), chromosome anomalies (7/13, 53.9%), orchitis (45/50, 90.0%), and cancer (3/6, 50.0%). Compared with the total SRR, the SRR of AZFc microdeletion (*p* = 0.029), Idiopathic (*p* = 0.0001), Cryptorchidism (*p* = 0.003), and Orchitis (*p* < 0.0001) had statistical differences (Table 2).

### 3.3. Histopathological Type and SRR

**Figure 2:**
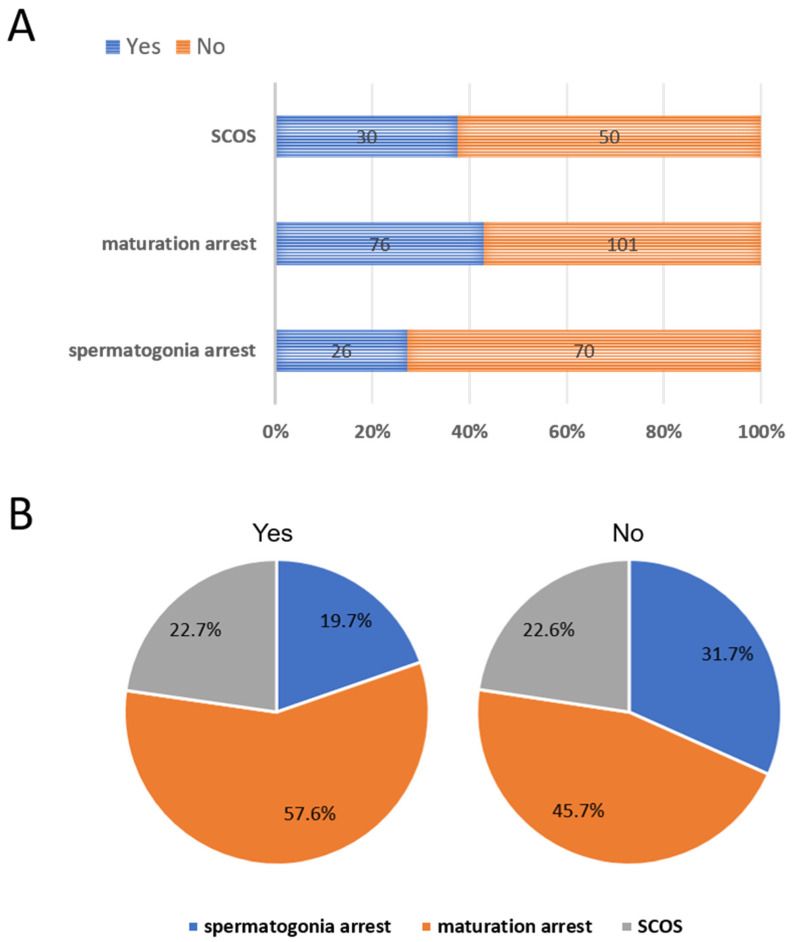
The sperm retrieval percentage by histopathological type. All patients were divided into 3 histopathological types present on pathology report at time of micro-TESE. (**A**) The sperm retrieval percentage by histopathological type in patients with NOA. (**B**) The proportion of each histopathological type in the successful sperm retrieval and unsuccessful sperm retrieval groups, respectively. SCOS, Sertoli cell-only syndrome. Yes, successful sperm retrieval. No, unsuccessful sperm retrieval.

Among the 627 patients who underwent the micro-TESE, 353 patients conducted the histopathological test. Three histopathological types were documented on pathology reports, including spermatogonia arrest, maturation arrest, and SCOS. Maturation arrest was the most common histopathological type in all cases. The second most common was spermatogonia arrest. The SRR were different for spermatogonia arrest (26/96, 27.1%), maturation arrest (76/177, 42.9%), and SCOS (30/80, 37.5%; Figure 2A). For those with successful sperm retrieval, maturation arrest was the most subject (76/132, 57.6%), followed by SCOS (30/132, 22.7%), whereas spermatogonia arrest was the least subject (26/132, 19.7%). Similarly, in the unsuccessful sperm retrieval group, maturation arrest was also the most subject (101/221, 45.7%), followed by spermatogonia arrest (70/221, 31.7%), whereas SCOS was the least subject (50/221, 22.6%; Figure 2B).

### 3.4. Clinical Pregnancy Association with the High-Quality Embryo Rate

ICSI and cumulative embryo transfer cycles were performed in 237 of the 247 couples with successful sperm retrieval. Of these, 172 couples achieved clinical pregnancies, 14 were lost to follow-up, and 76 achieved live births. The CPR was 77.1% (172/223), and the live birth rate was 44.2% (76/172) in these ICSI cycles (Figure 1). We found no differences in CPR among NOA etiologies (Table 3).

Among the ICSI treatments, the fertilization rate was 86.5 ± 1.1%, the cleavage rate was 97.4 ± 0.4%, and the high-quality embryo rate was 51.6 ± 2.0%. No difference in fertilization (87.2 ± 1.2 versus 83.8 ± 2.8) or cleavage rate (97.9 ± 0.4 versus 95.6 ± 1.3) was found between women with and without successful pregnancy. However, the high-quality embryo rate was higher in the successful pregnancy group (54.7 ± 2.2%) than in the unsuccessful pregnancy group (40.9 ± 4.6%; Table 4). We also analyzed the ICSI outcomes across the various etiologies, revealing no differences in fertilization rate, cleavage rate, or high-quality embryo rate among NOA etiologies (Table 5).

### 3.5. Clinical Pregnancy Association with the Female Partner’s Age

We compared the age, BMI, FSH, LH, PRL, E2, P, T, AMH, blood sugar, and thyroid-stimulating hormone (TSH) between women with and without clinical pregnancy. The age of the successfully pregnant women (28.99 ± 0.34 years) was significantly lower than that of the unsuccessful ones (30.92 ± 0.61 years). The groups were similar for all other parameters (Table 6).

A multivariable logistic regression model was used to identify predictors for the outcome of clinical pregnancy. The predictors, including female age, NOA etiology, BMI, and AMH, were analyzed in the multivariable regression model, and the forward stepwise (Likelihood Ratio) method was used to select the predictors. The final model included only one significant predictor: with increasing age, the risk for positive clinical pregnancy was reduced, with an OR of 0.921 (95% CI: 0.851–0.997, *p* = 0.043). NOA etiology was not significant for predictor (*p* = 0.084), although with increasing chromosome anomalies, the risk for positive clinical pregnancy was reduced, with an OR of 0.059 (95% CI: 0.005–0.751, *p* = 0.029; Table 7).

## 4. Discussion

This retrospective study aimed to analyze the SSR and clinical outcomes in NOA patients with various etiologies following micro-TESE and ICSI treatments. The results showed an overall SSR of 39.4%. The idiopathic NOA (INOA) group had the lowest SSR. The high-quality embryo rate differed between successful (54.7 ± 2.2%) and unsuccessful (40.9 ± 4.6%) pregnancies. Moreover, a negative association was found between the female partner’s age and clinical pregnancy. These findings suggested that NOA etiologies might be the principal factor affecting SSR, while the clinical outcomes were related to the high-quality embryo rate and women’s age.

Micro-TESE and ICSI are commonly applied to patients with NOA and are generally considered the standard methods. Just a few studies reported the SRR and clinical outcomes of patients with various NOA etiologies and sperm conditions after micro-TESE, and none reported the correlation between clinical pregnancy and fertilization or blastocyst development rate. Therefore, we analyzed the SRR, clinical pregnancy, and possible affecting factors following micro-TESE and ICSI for patients with NOA due to various etiologies and their female partners in a single center, aiming to provide relevant guidance for clinical diagnosis and treatment.

SRR is the most important parameter for patients with NOA. The overall SRR in our study was 39.4%, similar to the previously reported 40–50% [7]. Several candidate predictors for the SRR have been proposed, including the NOA etiology. Possible NOA etiologies include KS, YCMDs, cryptorchidism, idiopathic, orchitis, and cancer. AZFc microdeletion, a subset of YCMD, was associated with male infertility. AZFc microdeletion is the most commonly encountered YCMD, accounting for 60% of all YCMD deletions detected. The reported SRR of these patients when presenting with NOA is around 50% [10], similar to the 56.5% in our cohort. While no sperm can be retrieved by micro-TESE in patients with complete AZFa or AZFb deletions, those with AZFc deletions have the chance of some sperm retrieval [11]. KS is the most frequent chromosome disorder in males (1:650 newborn males), defined by a 47, XXY karyotype [12]. NOA and infertility occur in approximately 90% of those with KS [10]. The SRR for patients with KS in our study was 42.4%, in agreement with the findings in nonmosaic KS [13] and a meta-analysis that reported an SRR close to 50% in patients with KS [14]. However, Boeri et al. reported that the SRR was 21.4% in non-mosaic klinefelter pateients from five andrology centers [15]. Given the conflicting evidence in the literature on this topic, further large cohort studies are needed to corroborate the result. The highest SRR in our study was in patients with orchitis (90.0%), similar to the 100% SRR in a previous study [8].

Although several underlying causes are known, the exact etiology of most NOA cases remains unknown and is referred to as INOA. Interestingly, the lowest SRR in our study was in patients with INOA (27.6%). Chen et al. found that none of the INOA patients in their study had mature sperm, and they performed single-cell RNA sequencing (scRNA-seq) analysis and found that expression of CD164, LELP1, and TEX38 in germ cells was decreased and might be a genetic cause of INOA [16]. These studies suggest that genetic changes might be the main cause of INOA. More than 70% of NOA cases are INOA. The unknown pathogenesis and molecular basis might be the cause of the low SRR in these patients.

The SRR in men with NOA who are undergoing micro-TESE was associated with testicular histology, especially testicular histopathological heterogeneity [17]. In our study, maturation arrest has the highest SRR among three histopathological types. At the same time, the proportion of maturation arrest was the highest in both the successful and unsuccessful sperm retrieval groups. Hints that there is plenty of room for improvement in sperm retrieval.

The fertilization rate in our study was 86.5 ± 1.1%. Zhang et al. analyzed artificial oocyte activation performed on patients who successfully retrieved motile and immotile sperm by micro-TESE after ICSI. They found that immotile sperm injection with artificial oocyte activation displayed a high fertility rate (78.56%) [18], which was approximate to our result. The total CPR was 77.1% in our study, which is similar to the CPR in the other two studies, 80.04% [19] and 81.49% [20] from our hospital.

To our delight, we found that the NOA etiology was irrelevant to a successful clinical pregnancy outcome. We could conclude that once sperm retrieval from patients with NOA is successful, they all have an equal probability of having offspring. Although multivariable logistic regression analysis indicated that chromosome anomalies could be a predictor for clinical pregnancy, it may be caused by the small sample size. However, chromosome anomalies could affect embryonic development. Essers et al. reported that two-thirds of all pregnancy losses may be due to fetal chromosomal abnormalities [21]. However, our results demonstrated that the CPR was related to the high-quality embryo rate in our NOA cohort, similarly to a previous study in which the CPR was high when the top-scoring blastocysts in in vitro fertilization (IVF) or ICSI were transferred [22,23]. Furthermore, maternal age was negatively correlated with the CPR. This is reminiscent of a study in which the CPR was significantly lower in older women, even in those aged 31–35 at the time of IVF and embryo transfer (IVF-ET) [24]. Choi et al. also reported decreased CPR with increased maternal age following ICSI with testicular sperm from partners with azoospermia [25]. Yan et al. found no correlation between maternal age and the high-quality embryo rate in IVF-ET cycles [24]. Awadalla et al. reported that after controlling for morphology and the biopsy day, younger patients had higher sustained implantation rates for euploid embryos than older patients [26]. Decreased endometrial receptivity and thickness resulting from increased age are likely reasons for this observed reduction in the CPR [25,27,28]. To our knowledge, the current study was the first to report a trend of decreasing CPR with increasing age of the female partner of patients with NOA following micro-TESE and ICSI.

## 5. Conclusions

Our findings suggested that the SRR of patients with NOA undergoing micro-TESE was related to the NOA etiology. Although patients with orchitis had the highest SRR and those with idiopathic etiologies had the lowest, the NOA etiology was irrelevant to the clinical outcome. Moreover, we found a tendency to a decreasing pregnancy rate with increasing age of the patients’ female partners. Our findings could benefit formulating the recommendations given to the patients and the provision of suitable guidance for clinical work.

## 6. Limitation

However, our study had some limitations. The data were retrospectively analyzed, and the sample size of the included patients with NOA was limited. Prospective, large-sample, multicenter trials are needed to confirm our findings. Also, the techniques used to process the testicular samples may significantly affect the final sperm yield. The outcome measure utilized, SRR, does not encompass the goal of achieving live birth without birth defects. As some pregnancies are ongoing and some partners of patients with frozen retrieved sperm are still undergoing in vitro fertilization treatments, we could not present a cumulative live birth rate for the entire cohort.

## Figures and Tables

**Table 1:** Clinical characteristics of men with NOA who underwent micro-TESE.

Characteristic	Whole Cohort *n* = 627	Unsuccessful Sperm Retrieval (*n* = 380)	Successful Sperm Retrieval (*n* = 247)	*p*-Value
Age (years)	31.35 ± 0.19	31.11 ± 0.22	31.72 ± 0.36	0.656
BMI (kg/m^2^)	23.96 ± 0.14	23.83 ± 0.18	24.15 ± 0.23	0.275
FSH (IU/L)	21.37 ± 0.70	21.05 ± 0.87	21.83 ± 1.17	0.785
LH (IU/L)	9.84 ± 0.87	9.91 ± 1.34	9.73 ± 0.87	0.683
T (ng/mL)	5.08 ± 0.92	5.95 ± 1.54	3.81 ± 0.23	0.729
PRL (ng/mL)	15.48 ± 1.39	16.21 ± 1.88	14.40 ± 2.03	0.114
INHB (pg/mL)	43.92 ± 5.04	39.94 ± 7.61	49.10 ± 6.06	0.271
LTV (mL)	7.81 ± 0.19	8.05 ± 0.24	7.46 ± 0.29	0.165
RTV (mL)	7.87 ± 0.19	8.10 ± 0.25	7.54 ± 0.30	0.212

Data are presented as the mean ± SEM. Abbreviations: NOA, non-obstructive azoospermia; Micro-TESE, microdissection testicular sperm extraction; BMI, body mass index; FSH, follicle-stimulating hormone; LH, luteinizing hormone; T, testosterone; PRL, prolactin; INHB, inhibin B; LTV, left testis volume; RTV, right testis volume.

**Table 2:** Etiology and SRR of patients with NOA.

Etiology	*n* (%)	Successful Sperm Retrieval (*n*, %)	Association with All Patients *p*-Value
AZFc microdeletion	46 (7.3)	26 (56.5)	0.029 *
KS	85 (13.6)	36 (42.4)	0.637
Idiopathic	398 (63.5)	110 (27.6)	0.0001 ***
Cryptorchidism	29 (4.6)	20 (69.0)	0.003 **
Chromosome anomalies	13 (2.1)	7 (53.9)	0.391
Orchitis	50 (8.0)	45 (90.0)	<0.0001 ***
Cancer	6 (1.0)	3 (50.0)	0.685
Total	627	247 (39.4)	

Abbreviations: SRR, sperm retrieval rate; NOA, non-obstructive azoospermia; KS, Klinefelter syndrome; AZFc, Y chromosome azoospermia factor c. Compared with the total SRR, the SRR of AZFc microdeletion, Idiopathic, Cryptorchidism, and Orchitis had statistical differences. * *p* ≤ 0.05; ** *p* ≤ 0.01; *** *p* ≤ 0.001.

**Table 3:** Clinical pregnancy rate across the various etiologies.

Etiology	*n* (%)	Successful Pregnancy (*n*, %)	Association with All Patients *p*-Value
AZFc microdeletion	21 (9.4)	17 (81.0)	0.792
KS	34 (15.3)	30 (88.2)	0.180
Idiopathic	100 (44.8)	73 (73.0)	0.482
Cryptorchidism	18 (8.1)	13 (72.2)	0.575
Chromosome anomalies	5 (2.2)	2 (40.0)	0.088
Orchitis	43 (19.3)	36 (83.7)	0.422
Cancer	2 (0.9)	1 (50.0)	0.410
Total	223	172 (77.1)	

Abbreviations: AZFc, Y chromosome azoospermia factor c; KS, Klinefelter syndrome.

**Table 4:** ICSI outcomes in patients with NOA whose sperm was retrieved by micro-TESE.

Variable	Whole Cohort (%)	Successful Pregnancy (%)	Unsuccessful Pregnancy (%)	*p*-Value
Fertilization rate	86.5 ± 1.1	87.2 ±1.2	83.8 ± 2.8	0.399
Cleavage rate	97.4 ± 0.4	97.9 ± 0.4	95.6 ± 1.3	0.068
High-quality embryo rate	51.6 ± 2.0	54.7 ± 2.2	40.9 ± 4.6	0.008 **

Data are presented as the mean ± SEM. Abbreviations: ICSI, intracytoplasmic sperm injection; NOA, non-obstructive azoospermia; micro-TESE, microdissection testicular sperm extraction. ** *p* ≤ 0.01.

**Table 5:** ICSI outcomes across the various etiologies.

Etiology	Fertilization Rate	Cleavage Rate	High-Quality Embryo Rate
AZFc microdeletion	79.7 ± 5.1	98.9 ± 0.8	46.8 ± 5.8
KS	86.5 ± 2.7	96.6 ± 1.3	52.9 ± 4.9
Idiopathic	87.0 ± 1.7	96.8 ± 0.8	50.6 ± 3.2
Cryptorchidism	83.1 ± 4.7	98.8 ± 1.2	58.1 ± 7.1
Chromosome anomalies	88.8 ± 6.5	100.0 ± 0.0	39.0 ± 4.4
Orchitis	89.8 ± 1.5	98.1 ± 0.7	54.7 ± 4.4
Cancer	82.1 ± 12.1	92.9 ± 7.1	25.0 ± 25.0
*p*-value	0.858	0.443	0.537

Data are presented as the mean ± SEM. Abbreviations: AZFc, Y chromosome azoospermia factor c; KS, Klinefelter syndrome.

**Table 6:** Clinical characteristics of women with clinical pregnancy.

Characteristic	Whole Cohort *n* = 223	Successful Pregnancy *n* = 172	Unsuccessful Pregnancy *n* = 51	*p*-Value
Age (years)	29.43 ± 0.30	28.99 ± 0.34	30.92 ± 0.61	0.011 *
BMI (kg/m^2^)	21.83 ± 0.21	21.83 ± 0.21	21.68 ± 0.38	0.802
FSH (IU/L)	6.43 ± 0.15	6.34 ± 0.17	6.71 ± 0.34	0.300
LH (IU/L)	4.58 ± 0.27	4.30 ± 0.22	5.51 ± 0.95	0.060
PRL (ng/mL)	18.81 ± 0.85	19.09 ± 1.00	17.89 ± 1.55	0.456
E2 (pg/mL)	40.65 ± 1.62	38.91 ± 1.63	46.60 ± 4.38	0.241
P (ng/mL)	1.91 ± 0.30	1.93 ± 0.36	1.84 ± 0.55	0.158
T (ng/mL)	1.15 ± 0.33	0.83 ± 0.31	2.21 ± 0.95	0.054
AMH (ng/mL)	5.09 ± 0.26	5.36 ± 0.30	4.17 ± 0.43	0.067
BS (mmol/L)	4.92 ± 0.07	4.92 ± 0.08	4.94 ± 0.12	0.849
TSH (μIU/mL)	2.00 ± 0.07	1.98 ± 0.08	2.06 ± 0.19	0.888

Data are presented as the mean ± SEM. Abbreviations: BMI, body mass index; FSH, follicle-stimulating hormone; LH, luteinizing hormone; PRL, prolactin; E2, estradiol; P, progesterone; T, testosterone; AMH, anti-Müllerian hormone; BS, blood sugar; TSH, thyroid stimulating hormone. * *p* ≤ 0.05.

**Table 7:** Multivariable logistic regression analysis of predictors for clinical pregnancy.

Variable	β	S.E.	OR (95% CI)	*p*-Value
Age (years)	−0.082	0.041	0.921 (0.851–0.997)	0.043 *
Etiology				0.084
AZFc microdeletion	Reference			
KS	0.926	0.936	2.526 (0403–15.818)	0.322
Idiopathic	−0.368	0.611	0.692 (0.209–2.292)	0.547
Cryptorchidism	−0.808	0.785	0.446 (0.096–2.075)	0.303
Chromosome anomalies	−2.833	1.299	0.059 (0.005–0.751)	0.029 *
Orchitis	0.269	0.716	1.309 (0.322–5.329)	0.707
Cancer	−1.514	1.523	0.220 (0.011–4.351)	0.320

Abbreviations: AZFc, Y chromosome azoospermia factor c; KS, Klinefelter syndrome. S.E., standard error; OR, odds ratio; CI, confidence intervals. * *p* ≤ 0.05.

## Data Availability

The data that support the findings of this study is available from the corresponding author upon reasonable request.
